# The Role of Physical Activity in Post-COVID Schoolchildren’s Motor Competence

**DOI:** 10.1177/00315125251352661

**Published:** 2025-06-19

**Authors:** Roseany Silva, Giovanna Araújo, Daniele Licre, Maria Helena Silva, Pedro Ykaro Silva, Carolina Lima-Alvarez, Fábio Flôres

**Affiliations:** 1 Department of Physical Therapy, 28123 Universidade Federal do Rio Grande do Norte (UFRN), Natal, Rio Grande do Norte, Brazil; 2 28123 Nursing Technician Academic - School of Health (ESUFRN), 28123 Universidade Federal do Rio Grande do Norte (UFRN), Natal, Rio Grande do Norte, Brazil; 3 28123 Community Health Agent Technician - School of Health (ESUFRN), 28123 Universidade Federal do Rio Grande do Norte (UFRN), Natal, Rio Grande do Norte, Brazil; 4 70989 Universidade de Évora, Escola de Ciências Sociais, Évora, Portugal; 5 Centro de Investigação em Educação e Psicologia (CIEP), 70989 Universidade de Évora, Évora, Portugal; 6 Comprehensive Health Research Centre (CHRC), 70989 Universidade de Évora, Évora, Portugal

**Keywords:** competence, movement, children, skills

## Abstract

**Background:** Motor competence (MC) is closely linked to children’s physical activity (PA) levels, playing a critical role in their overall health and development. **Purpose:** This study explores the relationship between MC and PA among elementary school-aged children in northern Brazil. **Study Sample:** 261 children (52.11% boys and 47.89% girls) aged 5-14 (9.62 ± 2.70 years; 18.25 ± 3.92 Kg/m^2^). **Data Collection:** public and private schools in northern Brazil. **Research Design:** MC was assessed using the Motor Competence Assessment tool, and the PA levels were determined using the International Physical Activity Questionnaire (IPAQ) in its abbreviated form. **Results:** Most children (66.7%) were classified as physically active, with 27.2% showing very low levels of MC, and boys also outperformed girls. General results showed a moderate association between age and BMI (r = .402; *p* < .05), and a weak negative association between MC and BMI (r = - 0.177; *p* < .05). Also, a significant negative correlation was observed between BMI and MC in girls (r = −.361; *p* < .05). Multinomial regression analysis indicated that manipulative skills and overall MC significantly predicted higher PA levels. Higher levels of MC and increasing age were associated with greater PA and reduced odds of being irregularly active. **Conclusions:** These findings highlight the importance of early intervention, suggesting that enhancing MC can be a key strategy for promoting children’s health and fostering active lifestyles. In particular, the post-pandemic context reinforces MC as a critical factor influencing PA levels in children.

## Introduction

It has been well established in the literature that understanding the underlying mechanisms of physical activity (PA) is paramount to promoting a healthy lifestyle, especially in school-aged children. However, despite this knowledge, a concerning trend has emerged, where low levels of PA are associated with increased levels of overweight (Souza et al., 2010), enhanced metabolic disorders (Brambilla et al., 2011; Montesi et al., 2013; Pombo et al., 2023), poor social skills (Carson et al., 2019), and sleep problems (Watenpaugh, 2009) in this population. In this scenario, the COVID-19 pandemic significantly disrupted children’s daily routines, particularly by reducing opportunities for PA and limiting motor skill development (Lourenço et al., 2022; Pombo et al., 2023). Social distancing and school restrictions led to increased sedentary behaviors, especially screen time, which had long-term impacts on children’s physical health and motor competence (MC). Several studies have documented declines in motor skills following the lockdown, with children showing reduced proficiency in both gross and fine motor skills (Ayubi & Komainib, 2021; Kotzsch et al., 2025; Pombo et al., 2023; Pombo et al., 2020).

Stodden et al. (2008) proposed the spiral of engagement/disengagement, a conceptual model describing the relationship between PA and MC. In this model, the authors showed that PA provides a positive virtuous cycle of engagement, which leads to a strengthened association with MC over the developmental period. More specifically, as children engage in more PA, their MC improves, motivating participation in other PA and sports. Conversely, children with low levels of PA might engage less in movement experiences, leading to poorer MC. This lack of engagement discourages participation in sports and can perpetuate the cycle of disengagement and declining health. Ultimately, this cycle can result in negative long-term health outcomes as PA levels decrease further.

Since the spiral of engagement/disengagement model was suggested, much research has demonstrated positive associations between MC and PA (Burton et al., 2023; Logan et al., 2015; Utesch et al., 2019). These studies have consistently reinforced the importance of this reciprocal relationship. These investigations have confirmed Stodden’s model, showing a positive association between MC and PA among children and adolescents (Martinez-Lopez et al., 2024; Ortega-Benavent et al., 2024; Utesch et al., 2018). Pombo et al. (2023) showed that MC predicts children’s metabolic syndrome. The authors highlight that upper body strength is negatively associated with metabolic syndrome and positively correlates with children’s MC. Barnett et al. (2008) pointed out that adolescent object control proficiency was positively related to cardiorespiratory fitness. Additionally, Lopes et al. (2011) indicated that MC levels positively impact PA levels during childhood. Finally, positive linear relationships were observed between PA levels and total motor skills, particularly in object control and locomotor abilities (Barnett et al., 2024).

Despite the recommendation that children should spend at least 60 minutes/day in moderate-to-vigorous-intensity PA, few children meet the PA guidelines (Burns, 2024; Reis et al., 2024). The social distancing measures during the pandemic in recent years forced significant lifestyle changes in school-aged children, leading to a systematic decrease in their physical activity levels (Araujo et al., 2024; Pombo, Luz, Rodrigues, & Cordovil, 2020). Unfortunately, this pattern of reduced PA persists in their daily routines. Additionally, research has shown that the pandemic affected other aspects of children’s lives, such as increasing screen time (Kovacs et al., 2022; Schmidt et al., 2020). In addition, the literature highlights that MC levels declined after the lockdown, with boys experiencing an average decrease of 13 points in their global MC scores and girls a 16-point drop. The movement restrictions imposed during this period had a detrimental impact on the development of children’s MC (Pombo et al., 2021; Pombo, Luz, Rodrigues, Ferreira, & Cordovil, 2020). Specifically, Pombo and colleagues showed that the COVID-19 outbreak decreased children’s MC levels (Pombo et al., 2021) and negatively influenced their PA levels (Pombo, Luz, Rodrigues, Ferreira, & Cordovil, 2020). These findings suggest that the reduced PA experienced during the pandemic may have long-lasting effects on MC and health outcomes. Therefore, since improving PA levels through practice leads to enhanced levels of MC, it is imperative to analyze its relationship with school-aged children.

As far as we know, no other investigation has assessed the association between MC and PA levels among northern Brazilian children. This research gap is significant, given this region’s unique cultural and environmental factors. Based on the assumption that PA is associated with MC throughout life, the present investigation aims to evaluate whether the level of PA and biological factors are associated with MC after the pandemic outbreak. It is expected that age and MC will be positively associated and that boys will outperform girls regarding their levels of MC. In addition, we hypothesize that PA will influence MC levels.

## Materials and Methods

### Sample

The sample size was determined using G*Power v 3.1.9.7 software - Kiel University, Kiel, Germany (Faul et al., 2007), using the following parameters: Cohen’s effect size of 0.25 for correlation bivariate normal model, error probability α = 0.05, and β = 0.95. This calculation resulted in a sample size of 202 participants. Hence, 261 elementary school children between 5 and 14 years (age: 9.62 ± 2.70 years; BMI: 18.25 ± 3.92 Kg/m^2^) were conveniently recruited to participate in the investigation (see Table 1).

**Table 1.** Sample Characterization.

BMI (%)	Boys + girls	Boys	Girls
Underweight	33 (12.6)	14 (10.3)	19 (15.2)
Normal weight	147 (56.3)	81 (59.6)	66 (52.8)
Overweight	35 (13.4)	16 (11.8)	19 (15.2)
Obese	46 (17.6)	25 (18.4)	21 (16.8)

Participants (52.11% boys and 47.89% girls) were recruited from public (66.40%) and private (33.70%) schools in a city in Northern Brazil. The assessments were conducted at participating educational institutions and in the faculty laboratory under the same collection conditions. Data were collected after the pandemic outbreak (2022–2023).

The study’s inclusion criteria were limited to healthy children between the ages of 5 and 14 with no physical limitations or injuries. Additionally, none of the participants reported having COVID-19 during the year of data collection. Participants with developmental conditions or those who did not follow the procedures properly were excluded from the final sample.

### Procedures and Instruments

Oral assent was obtained from all children, and written consent was obtained from their legal guardians before the beginning of the tests. After agreeing to participate, legal guardians answered a screening form to characterize children and the International Physical Activity Questionnaire – short form (IPAQ-SF) regarding their children’s levels of PA. Children’s body mass index (BMI) was assessed using the following formula: BMI = BW/h^2^, where BMI (kg/m^2^) = Body mass Index; BW (kg) = Body weight; and h (m) = Height. Body weight and height were measured using a digital scale (SECA 761, Bacelar & Irmão Lda, Portugal) and a stadiometer (SECA 213, Bacelar & Irmão Lda, Portugal), respectively. These measurements were taken while participants were barefoot and wearing minimal clothing. To ensure the appropriateness of BMI classification for children and adolescents, the BMI was calculated using z-scores for each participant based on their age in decimal years and biological sex. These z-scores were then categorized according to WHO guidelines (WHO, 2007) as underweight (z < −2), normal weight (−2 ≤ z ≤ 1), overweight (1 < z ≤ 2), and obese (z > 2).

The children’s MC assessment utilized the Motor Competence Assessment (MCA) tool (Luz et al., 2016). Trained investigators administered the MCA, with each child taking about 20 minutes to complete the assessment. The MCA consists of six distinct tests: Jumping Sideways (JS) and Shifting Platforms (SP) (Stability component), Standing Long Jump (SLJ) and Shuttle Run (SHR) (Locomotor component), as well as Ball Kicking Velocity (BKV) and Ball Throwing Velocity (BTV) (Manipulative component).

In recent years, the MCA has been culturally adapted for use in Brazil (Sá et al., 2021), incorporating quantitative tests to reduce observational errors and avoiding a ceiling effect across developmental stages (Costa et al., 2024). The tool’s normative values converted individual test results into age- and gender-related percentiles (Rodrigues et al., 2019). The Stability, Locomotor, and Manipulative components were determined by averaging the percentile ranks of the two related tests in each category. Finally, the overall MCA score was calculated by averaging these three components (Rodrigues et al., 2019). Based on the total MCA score, MC levels were classified as low (≤30%), medium (31%–49%), or high (≥50%). Children were given a demonstration of the movements for each test, followed by a practice attempt. While they received motivational feedback, no specific test results were provided.

1. JS test: In this test, children were required to jump sideways over a 60 cm long, 4 cm high, and 2 cm wide wooden beam, using both feet simultaneously as many times as possible in 15 seconds. Points were awarded for each successful jump, and the best score from two attempts was recorded.
2. SP test: Children moved laterally for 20 seconds using two wooden platforms (25 cm × 25 cm × 2 cm). They received two points for each successful transfer, with one point per step taken, including passing the platform and moving onto it. The highest score from two attempts was recorded.
3. SLJ test: For this test, children jumped as far as possible from a standing position with both feet together. The distance from the starting line to the nearest heel at landing was measured in centimeters. The best performance from three attempts was used for analysis.
4. SHR test: Children ran towards a line 10 meters away, collected a wooden block, returned it past the starting line, and then repeated the process for a second block. The task was timed, and the fastest two attempts were used for scoring.
5. BKV test: Participants kicked a football (circumference: 64.0 cm; mass: 360.0 g) against a wall with maximum force. The speed of each kick was measured in meters per second using a Bushnell radar gun, and the highest speed from three kicks was recorded.
6. BTV test: Children threw a tennis ball (diameter: 6.5 cm; mass: 57.0 g) against a wall using an overarm throw with total effort. A Bushnell speed gun was used to measure the speed of each throw in meters per second, and the fastest of three attempts was noted.

Concerning the levels of PA, participants were classified following the IPAQ-SF (Matsudo et al., 2001), which the children’s legal guardians completed through an interview format conducted by trained researchers. To enhance reliability, researchers provided examples and clarified any questions during the administration process, and all responses were checked for internal consistency before inclusion in the dataset. Participants were classified as follows: very active (vigorous activity: ≥5 days/week and ≥30 minutes per session or vigorous: ≥3 days/week and ≥20 minutes per session + moderate and/or walking: ≥5 days/week and ≥30 minutes per session), active (vigorous activity: ≥3 days/week and ≥20 minutes per session; or moderate activity or walking: ≥5 days/week and ≥30 minutes per session; or any combined activity: ≥5 days/week and ≥150 minutes /without (walking + moderate + vigorous), irregularly active A (frequency: 5 days/week or duration: 150 min/week), irregularly active B (does not meet any of the criteria as mentioned earlier), and sedentary (one who does not practice any activity for at least 10 minutes during the week).

The university ethics committee approved the investigation (CAAE: 51565821.9.2004.5537) and followed all the guidelines of the Declaration of Helsinki (General Assembly of the World, 2014).

### Data Analysis

Descriptive analysis with mean and standard deviation was used to characterize data, but the Kolmogorov–Smirnov test did not confirm data normality; therefore, non-parametric tests were used. Spearman correlation was used to explore the relationships between MC and IPAQ. Correlation coefficients <0.30 were considered weak, those between 0.30 and 0.70 were considered moderate, and coefficients >0.70 were considered strong (Cohen, 2013; Field, 2005). The Mann-Whitney test was used to compare MC regarding sex differences. To perform the Multinominal Logistic Regression, the multicollinearity analysis was performed, and the association between the IPAQ, as a dependent variable, and the independent variables (locomotion, stability, manipulative, and MC; age and BMI) of the model was verified through the Nagelkerke R^2^ (adjusted) (the higher the R^2^, the better adjusted the model). Statistical Package for Social Sciences version 29.0 (IBM Corp, Armonk, NY, USA) was used, adopting an alpha significance level of 5%.

## Results

The descriptive analysis reveals that the study sample is balanced between the sexes (see Table 2). Our data also showed that 66.70% of the children are involved in PA regularly. Our results also indicated that most children, especially girls, are below the expected average regarding their MC levels.

**Table 2.** General Data of the Sample.

	Boys + girls	Boys	Girls
Practice physical activity regularly (%)			
Yes	174 (66.7)	101 (74.3)	73 (58.4)
No	87 (33.3)	35 (25.7)	52 (41.6)
Sports practiced (%)			
Soccer	54 (27.70)	52 (96.3)	2 (3.7)
Swimming	32 (16.40)	14 (43.8)	18 (56,2)
Karate	19 (9.70)	10 (52.6)	9 (47.4)
Volleyball	16 (8.2)	4 (25.0)	12 (75.0)
Ballet	14 (7.20)	0 (0.0)	14 (100.0)
Others	39 (30.8)	21 (53.8)	18 (46.2)
IPAQ (%)			
Sedentary	6 (2.30)	3 (2.20)	3 (2.40)
Irregularly active B	33 (12.60)	15 (11.00)	18 (14.40)
Irregularly active A	50 (19.20)	23 (16.90)	27 (21.60)
Active	133 (51.00)	68 (50.00)	65 (52.00)
Very active	39 (14.90)	27 (19.90)	12 (9.60)
Motor competence (%)			
Very low (<25%)	71 (27.20)	27 (19.90)	44 (35.20)
Low (25%–50%)	98 (37.50)	35 (25.70)	63 (50.40)
Average (50%–75%)	77 (29.50)	59 (43.40)	18 (14.40)
Above average (>75%)	15 (5.70)	15 (11.00)	—

Our results showed that the age sample wasn’t associated with most MC components (except stability). Age and BMI showed a moderate positive association, and BMI was negatively associated with MC.

Our results showed different trends when analyzing the associations controlled by BMI categories. The association analysis between BMI and MC revealed consistent patterns of negative associations, but none were statistically significant. Concerning MC components, only the stability in normal weight children showed significant, but weak negative associations. Age and BMI were negatively associated in normal weight and overweight children.

Our results showed that boys presented positive associations between age-manipulative components and BMI. On the other hand, girls only showed positive associations between age and BMI. Our findings also showed that girls presented a negative association between MC and BMI, suggesting that MC tends to decrease when BMI increases.

The comparisons between sexes showed significant differences between boys and girls when analyzing all the MCA tests separately, except for the JS test. When analyzing the MC, boys outperformed girls in all components. Nevertheless, BMI was similar between the sexes (Tables 3–6).

**Table 3.** Associations Between Age and MC.

	Age (years)	Stability (%)	Locomotor (%)	Manipulative (%)	MC (%)	BMI (Kg/m^2^)
Age (years)	1	−.333^*^	.010	.079	−.068	.402^*^
Stability (%)	—	1	.614^*^	.426^*^	.757	−.321^*^
Locomotor (%)	—	—	1	.613^*^	.888^*^	−.262^*^
Manipulative (%)	—	—	—	1	.842^*^	.067
MC (%)	—	—	—	—	1	−.177^*^
BMI (Kg/m^2^)	—	—	—	—	—	1

*p < .05.

**Table 4.** Associations Between Age and MC, Regarding BMI Classification.

BMI	Variables	Age (years)	Stability (%)	Locomotor (%)	Manipulative (%)	MC (%)	BMI (Kg/m^2^)
Underweight	Age (years)	1	−.356*	.003	.001	−.111	.125
	Stability (%)	—	1	.630*	.517*	.805*	−.234
	Locomotor (%)	—	—	1	.607*	.879*	−.225
	Manipulative (%)	—	—	—	1	.861*	−.203
	MC (%)	—	—	—	—	1	−.257
	BMI (Kg/m^2^)	—	—	—	—	-	1
Normal weight	Age (years)	1	−.364*	.017	.103	−.068	.533*
	Stability (%)	—	1	.563*	.401*	.751*	−.196*
	Locomotor (%)	—	—	1	.615*	.880*	−.066
	Manipulative (%)	—	—	—	1	.845*	.170*
	MC (%)	—	—	—	—	1	−.015
	BMI (Kg/m^2^)	—	—	—	—	—	1
Overweight	Age (years)	1	−.172	.147	.040	.024	.609*
	Stability (%)	—	1	.657*	.513*	.807*	−.273
	Locomotor (%)	—	—	1	.654*	.902*	−.067
	Manipulative (%)	—	—	—	1	.864*	−.009
	MC (%)	—	—	—	—	1	−.117
	BMI (Kg/m^2^)	—	—	—	—	—	1
Obese	Age (years)	1	−.054	.186	.069	.098	.037
	Stability (%)	—	1	.489*	.366*	.644*	−.279
	Locomotor (%)	—	—	1	.753*	.917*	−.279
	Manipulative (%)	—	—	—	1	.907*	−.065
	MC (%)	—	—	—	—	1	−.220
	BMI (Kg/m^2^)	—	—	—	—	—	1

*p < .05.

**Table 5.** Associations Between Age and MC Regarding Sex.

Sex	Variables	Age (years)	Stability (%)	Locomotor (%)	Manipulative (%)	MC (%)	BMI (Kg/m^2^)
Boys	Age (years)	1	−.205^*^	.135	.388^*^	.138	.473^*^
	Stability (%)	—	1	.626^*^	.384^*^	.779^*^	−.289^*^
	Locomotor (%)	—	—	1	.643^*^	.902^**^	−.186^*^
	Manipulative (%)	—	—	—	1	.805^**^	.166
	MC (%)	—	—	—	—	1	−.115
	BMI (Kg/m^2^)	—	—	—	—	—	1
Girls	Age (years)	1	−.532^*^	−.155	−.300^*^	−.386^*^	.320^*^
	Stability (%)	—	1	.511^*^	.325^*^	.725^*^	−.374^*^
	Locomotor (%)	—	—	1	.349^*^	.816^*^	−.432^*^
	Manipulative (%)	—	—	—	1	.731^*^	−.086
	MC (%)	—	—	—	—	1	−.361^*^
	BMI (Kg/m^2^)	—	—	—	—	—	1

*p < .05.

**Table 6.** Comparisons Between Boys and Girls.

Variables	Boys	Girls	U	Z	*p*
	Median (25th - 75th)	Median (25th - 75th)			
Age (years)	9.47 (7.65–11.80)	9.39 (7.43–11.80)	8259.00	−.396	.69
JS (%)	18.39 (5.89–38.06)	14.32 (6.03–32.99)	7740.50	−1.247	.21
SP (%)	24.79 (5.99–57.63)	11.18 (2.91–28.45)	6417.50	−3.418	.01
SLJ (%)	76.79 (42.54–92.91)	56.7 (29.62–79.21)	6033.50	−4.049	.01
SHR (%)	43.44 (14.68–75.23)	17.02 (4.94–40.08)	5482.00	−4.954	.01
BTV (%)	75.95 (41.64–97-65)	43.54 (18.31–67.66)	4913.50	−5.887	.01
BKV (%)	71.78 (25.31–89.66)	29.66 (8.96–56.23)	4949.00	−5.829	.01
Stability (%)	24.31 (8.68–48.26)	14.64 (6.20–29.65)	6723.50	−2.916	.04
Locomotor (%)	59.19 (32.37–78.96)	38.96 (19.76–55.45)	5464.00	−4.983	.01
Manipulative (%)	72.68 (37.87–90.53)	41.14 (18.35–54.79)	4497.00	−6.571	.01
MC (%)	52.68 (32.85–67-02)	33.88 (19.04–44.33)	4756.00	−6.145	.01
BMI (Kg/m^2^)	17.64 (15.50–20.17)	16.82 (15.45–20.02)	8133.50	−.602	.55

Our multinomial regression analysis investigates the impact of MC, age, and BMI on different PA levels. The analysis reveals that an increase in locomotor activity significantly decreases the odds of being classified under “Irregularly Active B” (*p* = .04). Conversely, increasing age significantly reduces the likelihood of being classified as “Irregularly Active A” (*p* = .00). Among the very active children, both manipulative component and MC appear to significantly increase PA levels (*p* = .04 and *p* = .03, respectively). Additionally, a significant positive relationship exists between age and higher PA levels in the active category (*p* = .03) (Table 7).

**Table 7.** Multinominal Logistic Regression.

IPAQ categories	Independent variables	Odds ratio (95% CI)	*p*
Sedentary	Stability	12.09 (0.87–16.74)	.32
	Locomotor	0.50 (0.00–56.07)	.77
	Manipulative	0.59 (0.00–3.59)	.18
	MC	0.17 (0.00–11.67)	.41
	Age	0.90 (0.64–1.27)	.55
	BMI	1.05 (0.85–1.30)	.66
Irregularly active B	Stability	5.01 (0.49–50.90)	.17
	Locomotor	0.09 (0.01–0.85)	.04
	Manipulative	3.33 (0.61–18.32)	.17
	MC	1.12 (0.18–7.19)	.90
	Age	0.86 (0.73–1.01)	.07
	BMI	1.07 (0.97–1.19)	.20
Irregularly active A	Stability	1.05 (0.15–7.68)	.96
	Locomotor	0.73 (0.13–4.26)	.73
	Manipulative	1.69 (0.41–6.99)	.47
	MC	1.34 (0.27–6.59)	.72
	Age	0.83 (0.72–0.95)	.00
	BMI	1.06 (0.96–1.16)	.25
Very active	Stability	0.54 (0.65–4.59)	.58
	Locomotor	1.73 (0.24–12.33)	.59
	Manipulative	5.66 (1.12–28.56)	.04
	MC	7.30 (1.30–41.36)	.03
	Age	1.19 (1.02–1.38)	.03
	BMI	0.89 (0.79–1.01)	.07

*Active was used as a reference.

## Discussion

Our main investigation goal was to evaluate the association of PA and MC. As expected, age and MC were positively associated in our sample, but only in girls (den Uil et al., 2023; Luz, Cordovil, et al., 2017; Rodrigues et al., 2019). Similar results were found concerning BMI and MC. Our findings also confirmed the hypothesis that boys would present higher levels of MC, confirming general results worldwide (Rodrigues et al., 2019; Sá et al., 2021). Regional or demographic factors that require further investigation may explain this divergence concerning boys and girls.

When analyzing the MC under sex, it was observed that boys outperformed girls in almost all tests. Notably, boys presented better results in all MC components. Similar results were found in the literature, showing that boys could perform those tests better than girls (Luz, Almeida, et al., 2017; Mačura & Sedlacek, 2021; Quitério et al., 2017; Rodrigues et al., 2019). Those differences may occur due to cultural and biological contexts in which boys and girls are inserted (Rodrigues et al., 2019), in which boys are encouraged to practice more sports and vigorous activities than girls (Flôres et al., 2021). This cultural encouragement might explain the observed higher MC scores in boys, suggesting a need for targeted interventions to promote gender equity in PA opportunities.

BMI was another critical variable in our investigation. According to Abarca-Gómez et al. (2017), there is an increase in the rate of obesity in children and young people over five years of age. In the present study, most children (56.3%) were normal weight, and an increased number of children were overweight (13.4%) or obese (17.6%), confirming the authors’ findings (see Table 1). Nonetheless, it is essential to highlight that our samples were composed of physically active children (66.70%), which could help to understand the variability of our findings. From this perspective, further studies should explore how different activity levels and nutritional factors contribute to these trends in northern Brazil. Table 3 shows a weak negative association between higher levels of MC and lower BMI levels. The literature supports this result. For example, Lima et al. (2023) conducted a systematic review that demonstrated that a higher BMI index in Brazilian children has a negative impact on MC. Also, higher BMI indexes were shown to be negatively associated with lower levels of motor skills performance.

Although previous studies have reported a decline in PA with increasing age (Nyberg et al., 2009), and global estimates suggest that 80% of children are insufficiently active, with higher inactivity rates among girls (85%) compared to boys (78%) (Guthold et al., 2020; Hallal, 2012), our findings did not align with these trends. In our sample, most children were classified as physically active (see Table 3 for further details), and boys were more active than girls. This apparent contradiction may be explained by the unique post-pandemic context in which our data were collected. Following prolonged school restrictions on social interaction due to COVID-19, children may have shown an increased motivation to return to physical activities, possibly as a form of recovery from the effects of confinement. Recent evidence indicates that extended lockdowns had a detrimental impact on children’s MC when compared to pre-pandemic levels (Cumilef-Bustamante et al., 2023; Pombo et al., 2021), and our study offers further insight into how these effects may vary by region and context.

Furthermore, although we initially expected a positive association between age and MC based on existing literature, this was not observed in the overall sample. However, when stratified by sex and BMI category, more diverse patterns emerged, such as positive associations between age and manipulative skills in boys, and negative associations between age and MC components in girls. These findings suggest that broader sociodemographic and environmental factors, such as disparities in access to adequate space, equipment, and instruction between public and private schools (Ferreira et al., 2023; Queiroz et al., 2016), may influence the relationship between age and MC. Thus, the absence of a general age-MC association in our sample likely reflects the complex interplay between post-pandemic recovery, socioeconomic conditions, and gender-based differences in motor experiences.

The multinomial regression analysis explored how MC impacts PA levels among 5- to 14-year-old children. Results indicated that better manipulative competence and overall higher MC scores are associated with an increased likelihood of children being very active. Pereira (2018) observed that MC can predict higher levels of PA during childhood. Thus, these findings underscore the importance of motor skill development in increasing PA levels and promoting physical education programs to foster essential motor skills from an early age. This highlights the role of interventions focusing on skill acquisition to encourage lifelong engagement in PA.

Nevertheless, it is essential to highlight some limitations found in this investigation. Firstly, the lack of evaluation of the specific types of sports the children practiced may have limited our ability to understand the degree of their PA levels fully. Additionally, a more detailed analysis of socioeconomic factors, particularly the distinction between public and private school students, could provide deeper insights.

Our findings may significantly affect public health policies and educational strategies to promote PA and develop MC among children. The investigation suggests the necessity for gender-specific programs to address disparities in PA and motor skills development between boys and girls. Also, schools, particularly in underserved areas, must prioritize integrating diversified and developmentally appropriate physical activities into their curricula. Therefore, our results highlight the role of educational institutions in nurturing MC through structured physical education classes, which could be important in counteracting the trends of inactivity and obesity in children. Future policies should consider addressing these gaps and focusing on PA and MC to ensure holistic child development.

## Conclusion

This investigation provides valuable insights into the relationship between PA and MC in school-aged children from northern Brazil, emphasizing the critical role MC plays in promoting active lifestyles, especially in the post-pandemic context. Our findings revealed significant sex-based disparities, with boys consistently outperforming girls in all MC components, and a weak negative correlation between MC and BMI, highlighting the impact of weight on development even in physically active children. Notably, manipulative skills emerged as a key predictor of higher PA levels, underscoring the importance of targeted motor skill development in encouraging sustained engagement in PA. Overall, this investigation contributes to the growing evidence supporting early interventions that enhance MC to improve children’s health outcomes and promote lifelong PA.
